# Nephrolithotomy

**Published:** 1890-09-20

**Authors:** 


### NEPHROLITHOTOMY.

Mr. Jacobson, assistant surgeon to Guy's Hospital, recently contributed a lengthy and exhaustive paper on the symptoms and conditions which justify nephrolithotomy (*B.M.J.*). First, there is continued hæmaturia, or passage of blood and pus. But other causes than stone in the kidney pelvis may cause hæmaturia, such as the passage of uric acid crystals, tubercular kidney, granular kidney growths, &c. There is also an endemic hæmaturia, especially in the East, to which the author does not refer, and which has been theorised to be caused by malaria or by filaria in the blood. Secondly, pain and tenderness in the lumbar region or elsewhere. The pain caused by a kidney stone is usually dull and gnawing, sometimes radiating to the testes and sciatic nerve. Although there may be no tenderness the patient will complain of a sharp stabbing pain or percussion which is very characteristic. Thirdly, points in the previous history. These are chiefly lithiasis, oxaluria, history of a previous stone, or of a previous colic. Fourthly, frequency of micturition. The co-existence of irritability of the bladder with renal calculus is well known, and may be explained either by nerve disturbance, or by the blood and pus or the over acid urine which often accompanies stone in the kidney. Fifthly, absence of any condition in the rest of the genito-urinary tract which will explain the symptoms. Sixthly, failure of previous treatment to give relief. The chief practical points in the performance of nephrolithotomy are to count the ribs, so that an opening into the pleural cavity may be avoided; to make a sufficiently free incision by converting the usual lumbar incision into a T-shaped one; to pack the colon away with sponges, as it is often troublesomely distended with flatus; if the stone cannot be felt, the kidney should be drawn out of the wound as far as possible; in puncturing a kidney to try to open the calyces systematically. If palpation and acupuncture fail to find a stone the kidney should be carefully opened and sounded. Hæmorrhage is certainly arrested by firm, careful plugging with strips of sal alembroth gauze. The sources of difficulty in removing a stone are principally a very mobile kidney, which gets away deep in the wound; a stone situated on the anterior surface, and near the entrance of the vessels; a small stone in a sacculated kidney, the stone falling into one of the saccules, and thus being hard to find; multiple calculus, especially in a suppurating damaged kidney. If the kidney has been much disturbed during the operation it should be stitched *in situ*.

